# Cardiac autonomic functions and the emergence of violence in a highly realistic model of social conflict in humans

**DOI:** 10.3389/fnbeh.2014.00364

**Published:** 2014-10-21

**Authors:** Jozsef Haller, Gabriella Raczkevy-Deak, Katalin P. Gyimesine, Andras Szakmary, Istvan Farkas, Jozsef Vegh

**Affiliations:** ^1^Behavioral Neurobiology, Institute of Experimental Medicine, Hungarian Academy of SciencesBudapest, Hungary; ^2^International Training CentreBudapest, Hungary

**Keywords:** heart rate, heart rate variability, social conflict, violence, new paradigm, autonomic functions, arousal

## Abstract

Among the multitude of factors that can transform human social interactions into violent conflicts, biological features received much attention in recent years as correlates of decision making and aggressiveness especially in critical situations. We present here a highly realistic new model of human aggression and violence, where genuine acts of aggression are readily performed and which at the same time allows the parallel recording of biological concomitants. Particularly, we studied police officers trained at the International Training Centre (Budapest, Hungary), who are prepared to perform operations under extreme conditions of stress. We found that aggressive arousal can transform a basically peaceful social encounter into a violent conflict. Autonomic recordings show that this change is accompanied by increased heart rates, which was associated earlier with reduced cognitive complexity of perceptions (“attentional myopia”) and promotes a bias toward hostile attributions and aggression. We also observed reduced heart rate variability in violent subjects, which is believed to signal a poor functioning of prefrontal-subcortical inhibitory circuits and reduces self-control. Importantly, these autonomic particularities were observed already at the beginning of social encounters i.e., before aggressive acts were initiated, suggesting that individual characteristics of the stress-response define the way in which social pressure affects social behavior, particularly the way in which this develops into violence. Taken together, these findings suggest that cardiac autonomic functions are valuable external symptoms of internal motivational states and decision making processes, and raise the possibility that behavior under social pressure can be predicted by the individual characteristics of stress responsiveness.

## Introduction

Autonomic functions received much attention in recent years as biological correlates and predictors of aggressiveness under social pressure. On one side, the neurovisceral integration model posits that heart rate variability (HRV), is an indicator of the functional integrity of the neural networks implicated in emotion-cognition interactions (e.g., prefrontal-subcortical inhibitory circuits), and phasic HRV increases were associated with self-regulatory effort (Thayer and Lane, 2000; Park and Thayer, 2014). On the other side, resting heart rates (HR), autonomic reactivity, and HRV are recognized as important determinants of antisocial behavior and aggressiveness (Lorber, 2004; Ortiz and Raine, 2004; Patrick, 2008; Scarpa et al., 2010). According to these two converging lines of evidence, HR and HRV are suggestive external symptoms of internal decision-making processes on one side, and aggression proneness on the other side. Despite efforts, however, the triple association between autonomic functions, decision making and aggressiveness remains poorly understood. One of the seldom noticed problem of this area is that the study of autonomic indicators on one side and aggressiveness and aggression-related decisions on the other side are either temporally segregated or the latter two phenomena are studied in artificial laboratory conditions, where aggressiveness is symbolic or mild.

One line of research addresses the above association based on the history of subjects, who did perform real acts of aggression in the past (e.g., they committed criminal offense), but were peaceful when autonomic measurements were performed; moreover, they readily cooperated with the study personnel (otherwise the measurements could not be performed). Such studies showed that a history of antisocial behavior correlates with basal HR and HRV, as well as with autonomic responsiveness, and the nature of the correlation depends on the type of aggression performed and with psychological mechanisms that may underlie the association. For instance, sensation seeking was proposed as a possible outcome of low resting HR that may lead to aggressive tendencies (Wilson and Scarpa, 2011); resting HR was high, while resting HRV was low in subjects showing reactive aggression, while proactive aggression was associated with high HRV (Scarpa et al., 2010); blunted HR reactivity was associated with proactive, while exaggerated reactivity with reactive aggression (Murray-Close and Rellini, 2012). The common characteristic of such studies is that they investigate the association between real forms of aggression performed in the past with actual measures of autonomic function; i.e., they leave open the question of how behavior and autonomic functions correlate while aggressive acts are actually performed.

Another group of studies—usually more concerned with stimulus evaluation and decision making—investigates autonomic functions and aggressiveness in parallel, but the latter is either symbolic as in the case of violent video games (Ivarsson et al., 2009; Stephens and Allsop, 2012), or mild as in the case of the Taylor Aggression Paradigm, where mild punishments (e.g., air blasts) are delivered to non-visible, fictional opponents (Verona and Sullivan, 2008; Ward et al., 2008). Similarly mild procedures are employed when the interactions between aggression promoting factors and autonomic functions is studied. e.g., threats are verbally conveyed by a third person or are represented by pictures (Williams et al., 2003; Ward et al., 2008; Grasso and Simons, 2012), emotionality and anger are usually studied in simulated situations (Stephens and Groeger, 2011; Barhight et al., 2013) etc. Such studies reveal acute interactions between cognitive-behavioral processes and autonomic functions, but fail to address behaviors of societal concern i.e., real forms of aggression.

Studies into the cognitive and behavioral aspects of human aggression are curbed by ethical and technical constraints. As such, studying mild forms of aggression in the laboratory is only natural. We present here, however, a model that overcomes the difficulty of studying real forms of aggression in parallel with autonomic recordings. Particularly, we report on findings obtained in the International Training Centre (Budapest, Hungary), where police officers originating from a large number of countries are trained to perform operations under extreme conditions of stress. Subjects were exposed to highly realistic training situations (“incidents”), where they showed a wide repertoire of real aggressive acts while their cardiac autonomic functions were recorded. To investigate the impact of arousal on decision-making and aggressive behavior, a conflict was created between the valence of antecedents and incidents: a primarily aggressive training context was preceded by peaceful activity, while a primarily peaceful context was preceded by the induction of aggressive arousal. We hypothesized that antecedents will have a strong impact on arousal levels, and this will influence behavior to a greater extent than information available in the training arena.

## Materials and methods

### The study site

The study was performed in the International Training Centre (ITC, Budapest, Hungary), which is run by the Ministry of Interior of Hungary and hosts 4 international police academies (ILEA, MEPA, CEPOL, and ICOFI). Law-enforcement officers from over 90 countries study in the academies (for details see http://www.nokitc.hu/). The mission of the special training method presented here is to prepare police officers to perform operations under extreme and stressful situations, and was created, patented and accredited by the training team that consists of psychologists, police tactical officers and education experts.

### Participants

Study participants were physically and psychologically healthy, active, seasoned police officers who have not been trained at the ITC earlier i.e., they were naive to the training procedures employed. The age range was 25–35 years. Sample size was 164 (19 females, 11.6%). Heart rate recordings were obtained from 119 subjects. The officers came from over 10 countries. Participation was voluntary, and participants gave their informed consent to both their involvement in the training program and the recording of their behavior and autonomic functions. Personal data were not recorded and performance during the training session was not reported to superiors. Participants were inherently made unrecognizable on the recordings by masks that protected them from training handguns (see below).

Two highly experienced police tactical officers acted as suspects in the training situations presented below. Those authors of the present study who are not employees of ITC and were not involved in the training procedure (JH, GD, GPK) note that their acting skills are exceptional, and enhanced considerably the naturalistic character of the training.

### Training procedure

Officers (typically 8 at a time) arrived to the training site early in the morning (8 am), and were given a briefing by a trained psychologist (JV). The briefing started by general, relaxing questions (e.g., “How do you feel here”), and was followed by a general description of the training procedure including the review of the rules to be observed, and safety instructions. The details of the training situations were not disclosed. After the briefing, the heart recording device (Polar S810i, Polar Oy, Finland) was secured to the chest of subjects, who were also provided with protective equipment (face mask and throat protector). They received Glock 17T training handguns with Simunition FX ammo (General Dynamics-Ordnance and Tactical Systems-Canada Inc., Canada) which contains hollow plastic projectile filled with dyed soap.

Groups of two partners were formed; due to their small number, female officers were paired with male partners except for one case. The first pair was instructed that the police had received information from neighbors about strange noises in a flat, the owners of which were on vacation i.e., they should not have been home. The pair was assigned the job of investigating the case and moved to the training site adjacent to the lecture hall (for a description see below). This pair always found two burglars in the flat, which after an initial phase of diversion attacked them (the incident is described below).

The rest of officers remained in the lecture hall, from where they were able to hear the noises of the incident (shouting, throbbing caused by physical fights, and shootings). After the first pair had finished the task, it was returned to the lecture hall by the training supervisor with the words “Here I bring you what remained of your colleagues.” These words together with the noises heard deliberately aimed at increasing aggressive arousal in the second pair, which was submitted to the training situation immediately after the return of the first pair. They were instructed in the same way, and received the same task i.e., to investigate the source of noises heard by a neighbor in a temporarily deserted flat. They were moved to the same but slightly rearranged flat, where they found two international journalists who became nervous about their intrusion, but readily identified themselves, and tried to contact their embassies (see below).

Assignment to the two training situations was random. The remaining two pairs were involved in tasks other than the ones present here. By the end of the whole training session (the four different “incidents”), the supervisor (IF) presented the video and heart rate recordings made during the incidents, and interactively discussed them with the officers as part of their debriefing. JH (not an ITC employee) found that the atmosphere of these discussions was very relaxed; the officers did not consider the open presentation of their recordings offensive by any means; moreover, they were able to laugh about their own mistakes, and considered the debriefing very instructive for improving their own and their colleagues' performance in real-life situations.

*The training enclosure* consisted of a corridor with three doors which lead into three flats. The door of the flat to be investigated was half-opened; the rest of the doors were closed. Behind the closed doors, “neighbors” were residing who could be asked for information. The flat to be investigated comprised an anteroom, a kitchen, a bedroom and a bathroom; each of the latter three was connected to the anteroom. Rooms were furnished appropriately. The behavior of study subjects was video recorded from above by means of video cameras that covered the whole training site including the corridors.

#### Incident 1 “burglars”

The flat was arranged such to make the officers believe the interception of a burglary. The cupboard of the anteroom was opened, half-empty, and various items were scattered over the floor including clothes, computer spare parts (e.g., a keyboard) or a VCR. Suspect A was not visible in the initial phase of the incident, but came out to the anteroom shortly after the officers entered the flat. He looked drunk and started asking questions in raised voice (“Who are you?,” “What are you doing here?”). Upon interrogation, he avoided identifying himself, declined to account for his presence in, and was unaware of the owner of, the flat. He was verbally aggressive, and defended himself physically if attacked. Suspect B was hiding such that none of the officers of this study noticed his presence. After about 3 min, he ran out with a gun in hand and started shooting at officers. Concomitantly, Suspect A also attacked.

#### Incident 2 “journalists”

The flat was arranged such to make the officers believe that people are moving in. The cupboard of the anteroom was open and empty, and there was a luggage on the bed that contained carefully packed clothes. Suspect A was not visible initially but came out shortly after the officers had entered the anteroom. He started asking question in raised voice (“Who are you?,” “What are you doing here?”). He did not look drunk, readily identified himself as an international journalist (showed up his journalist card), and tried to contact his embassy by phone. He was verbally aggressive, and defended himself physically if attacked. Suspect B was located in the bathroom and after about 3 min he came out with a video camera in hand, and started recording the scene.

### Behavioral recordings

The ethological profile of subjects during the incidents was drawn by means of non-verbal behavior analysis. Behaviors were not interpreted as “correct” or “incorrect” from the point of view of police regulations, i.e., scoring was factual.

In both incidents, action was divided into three phases. The *opening phase* started by the supervisor when subjects entered the corridor and ended when the officers entered the flat. During this period, the following behaviors were observed (in their usual order of occurrence): *verbal orientation* (asking the neighbor), *visual orientation* (inspecting the corridor, listening at the door, or looking into the flat without entering), *communication between officers* (discussing the action, assigning roles), *communication with headquarters* (through walkie-talkie), *communication with suspects* (questions, calls, and warnings shouted through the half-opened door), and two types of behaviors called together *uncertainty*: inactivity (standing around the door without obvious activity), and hesitation (going halfway through, and standing in the door). The duration of behaviors and the total duration of the opening phase were recorded. We also recorded whether subjects held their guns in hand before entering the flat. Because this can be considered a preparation for the next phase, data will be presented together with those recorded there.

The *action phase* started when the officers entered the flat and ended when officers surrendered or prevailed in conflict (see below). The following behaviors were observed: *procedural activities* (inspecting the flat, securing doors, interrogating subjects, etc.), *verbal control* (commands like “Stay put,” “Turn around,” etc.), and *aggressive acts*. The latter were divided into *provoked* (responses to aggression initiated by suspects) and *non-provoked* (self-initiated). Within both categories, *verbal* (shouting, verbal threats) and *physical aggression* was differentiated (pushing, hitting, kicking, wrestling, and holding down). *Gun use* was also observed, and classified as provoked when it was prompted by physical aggression by suspects (armed or not) and non-provoked when it was initiated by subjects in the absence of obvious threats by suspects.

The *end phase* started when officers surrendered or prevailed in conflict. Surrendering by officers covered the following behaviors: allowing suspects to leave the flat, being pushed out of, or closed into the flat, being disarmed, laid on the floor or handcuffed. Officers were considered *surrendering* if they showed any of these behaviors. They were considered *non-surrendering* (“prevailing”) when durably obstructed the above-mentioned actions by suspects, managed to physical control them (laid down or handcuffed suspects) or shot them “dead.” The length of this period depended to a large extent on the context. The supervisor usually stopped the action when either surrendering or reluctance to surrender became obvious or when officers lost control (e.g., they became excessively aggressive). In some instances of surrender (e.g., when suspects left the flat), the action was allowed to develop for up to 9 min, to see how the officers handled the situation. Due to the widely different time allowed to this phase, the duration of behaviors was not recorded; instead, officers were characterized as surrendering or non-surrendering based on the criteria described above.

### Cardiac autonomic function

Autonomic activity was recorded by a validated portable device (Polar S810i, Polar Electro Oy, Finland) (Gamelin et al., 2006; Porto and Junqueira, 2009). The device registers R-R intervals of all heartbeats, based on which HR (beats/min) and HRV (root mean of the squared successive differences, r-MSSD) were calculated, the latter indicating variation in the time interval between heartbeats. Albeit several approaches are employed to characterize this aspect of cardiac activity, the r-MSSD method is widely used in studies that address stress and arousal effects and consequently, the application of this measure ensured comparability with other studies, including those referred to in this paper (Hansen et al., 2003; Ruiz-Padial et al., 2003; Ivarsson et al., 2009; Porto and Junqueira, 2009; Scarpa et al., 2010; Krypotos et al., 2011). Recordings were divided into the following periods: the *opening period* (corresponds to the similarly named phase of the training session), *the non-aggressive period of the action phase* (corresponds to the inspection, inquiry, and communication periods of the action phase), and *the aggression period of the action phase* (marked by the outbreak of physical aggression). Noteworthy, physical aggression was present in all the trials where autonomic functions were recorded. The opening period and the non-aggressive period of the action phase lasted on average 2 and 3 min, respectively. For the aggression period, the first 3 min of the recordings were shown, because its duration was highly variable and only 3 full min were available for all encounters. Each min of the aggression period was analyzed separately. The coverage of the end phase was irregular for the reasons shown above; therefore, autonomic activity was not evaluated for this phase.

### Data analysis

Data are shown as mean ± the *SE* of the mean. Behaviors were compared by Kruskal-Wallis ANOVA. Frequencies (e.g., the share of gun using officers) were analyzed by cross-tabulation. HR and HRV observed in the two incidents were compared by two-factor ANOVA (Factor 1: the incident; Factor 2: the periods of incidents as presented above). The study of the interaction between autonomic activity and behaviors focused on gun use and surrender, two critical behaviors from the perspective of both aggressiveness and police action. In this respect, HR and HRV were analyzed by three-factor ANOVA; Factor 1 was the incident (levels: Incident 1 and 2), Factor 2 was behavior (levels: behavior present or absent), while Factor 3 was time (levels: the periods of the incidents as presented above). Gender effects were not investigated due to the small number of female participants. The visual inspection of recordings showed that females took initiative in about half of the trials where they were present; in addition, no obvious gender differences were observed in any of the behaviors performed. For this reason, findings in the two genders were analyzed together. The country of origin was not used as an ANOVA factor for the same reasons. Subjects came from a variety of countries, the representation of which in the sample was highly different. In addition, nationality did not seem to have an impact on behavior and autonomic functions. Multiple Regression analysis was used to evaluate the relationship between behavior and autonomic functions.

## Results

### Overall evaluation of behavior

Although actions were performed within the framework of a training session, subjects showed genuine acts of aggression during the trials. All subjects exchanged spirited verbal threats with suspects, all but 2 (97.5%) engaged in physical fights (delivered blows, kicked and/or wrestled), and 37% shot the suspects. The realistic nature of aggressive interactions was evident also in the end phase, when subdued officers resumed normal behavior rather slowly, despite the fact that suspects never hit or kicked them, and the aggressive phase never lasted longer then a few minutes, i.e., exhaustion could not play a role. Noteworthy, “suspects” (i.e., training officers) were highly experienced in physical fighting; they diverted practically all blows and kicks directed at them, and never lost control. As a consequence, nobody was injured in fights, despite the highly realistic nature of these.

### Behavioral differences in incidents 1 and 2

**Figure 1:**
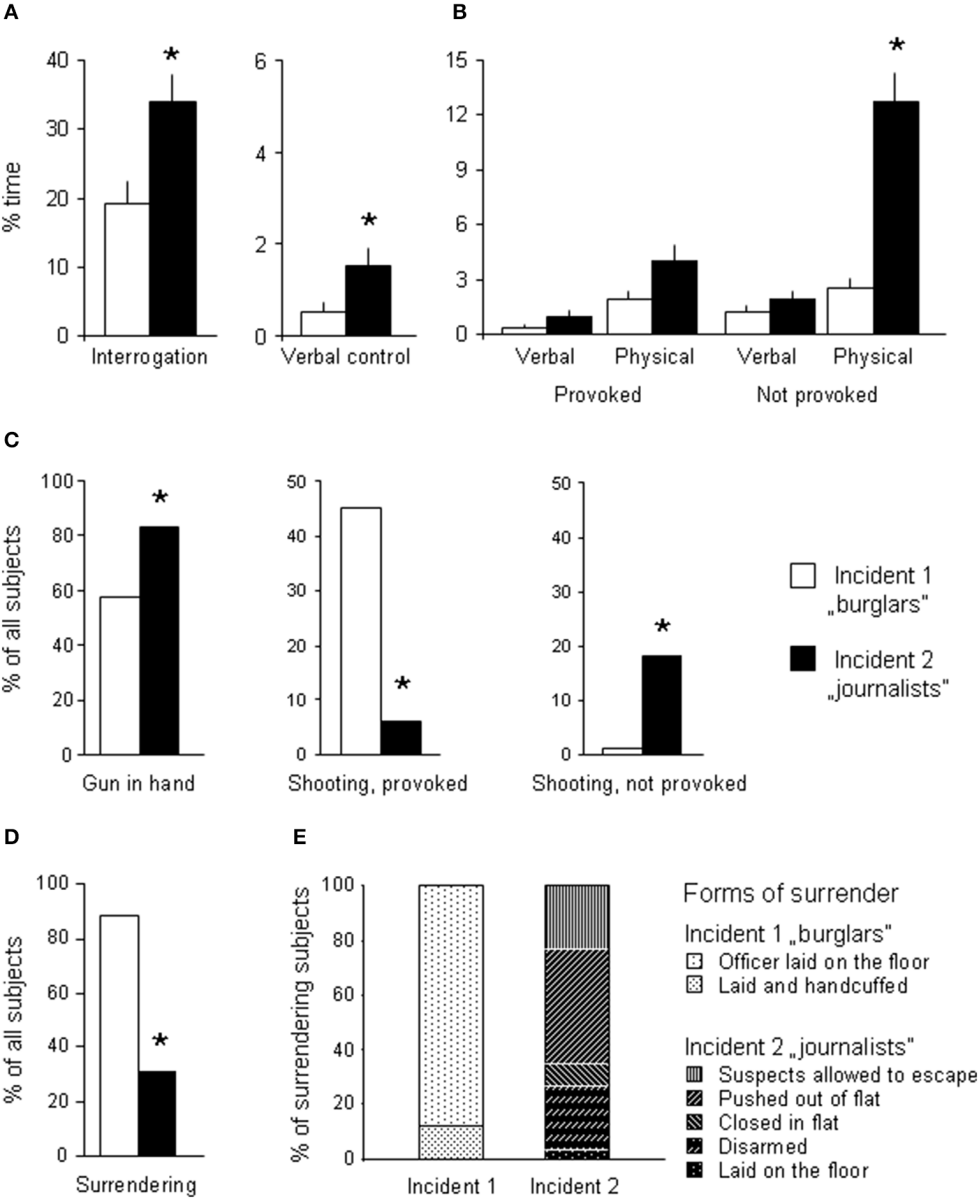
**Behaviors shown during the action and end phases**. For behaviors shown during the opening phase see Table 1. **(A)** Behavior in the non-aggressive period of the action phase; **(B)** behavior in the aggression period of the action phase; **(C)** Gun use: gun held in hand when entering the flat, and the share of officers who shot suspects in response to or without provocation; **(D)** the share of surrendering subjects; **(E)** the share of various forms of surrender. ^*^significant differences between incidents (*p* < 0.05 at least).

No behavioral differences were observed in the *opening phase* (Table 1). In the *action phase*, the officers of Incident 2 spent significantly more time with procedural activities [*H*_(1, 80)_ = 8.79; *p* = 0.003] and verbal control [*H*_(1, 80)_ = 5.33; *p* = 0.02] (Figure 1A). Verbal aggression did not differentiate the two incidents [*provoked verbal aggression*: *H*_(1, 80)_ = 0.81; *p* = 0.4; *non-provoked verbal aggression*: *H*_(1, 80)_ = 1.37; *p* = 0.3] (Figure 1B). Differences in provoked physical aggression were not significant [*H*_(1, 80)_ = 1.21; *p* = 0.3]. By contrast, non-provoked physical aggression was dramatically increased in Incident 2 (journalists) as compared to Incident 1 (burglars) [*H*_(1, 80)_ = 34.19; *p* < 0.0001] (Figure 1B). In Incident 2, a larger proportion of officers held guns in hand while entering the flat (Chi square = 8.14; *p* = 0.0043) (Figure 1C, left). Quantitatively, gun use was similar, but circumstances differed widely. In Incident 1, gun use was provoked in all cases but one. In Incident 2, gun use was non-provoked in all cases but 3. Differences were highly significant (*provoked gun use*: Chi square = 32.77; *p* < 0.00001; *non-provoked gun use*: Chi square = 13.57; *p* = 0.0002). In the *end phase*, readiness to surrender was significantly different (Chi square = 53.65; *p* < 0.00001) (Figure 1D). The forms of surrender showed almost no overlaps (Chi squares for particular forms of surrender were above 30, while *p*-values were below 0.001 at least) (Figure 1E).

**Table 1:** **The behavior of subjects in the opening phase**.

**Incident**	**Total duration**	**Orientation**		**Communication with**			**Uncertainty**	
		**Verbal**	**Visual**	**Partner**	**Headquarters**	**Suspects**	**Inactivity**	**Hesitation**
1(“burglars”)	125.2 ± 22.2	7.8 ± 2.4	8.2 ± 2.3	4.8 ± 1.4	23.5 ± 3.9	17.4 ± 2.8	31.9 ± 3.1	5.9 ± 1.1
2(“journalists”)	109.1 ± 17.6	10.4 ± 2.9	8.9 ± 2.5	4.5 ± 1.0	27.9 ± 3.6	13.7 ± 2.6	30.8 ± 3.6	3.4 ± 0.5
*H*_(1, 80)_	0.01	0.35	0.15	0.01	0.77	0.45	0.18	1.14
*p*	0.9	0.5	0.7	0.99	0.4	0.5	0.6	0.3

*Data (mean ± s.e.m.) are shown in seconds for total duration and as % time of total duration for particular behaviors*.

Thus, subjects submitted to Incident 2 showed high levels of non-provoked aggression, although suspects were in no violation of the law. Mainly provoked aggression was seen in Incident 1, where suspects seemed to commit a criminal offense.

### Autonomic functions

**Figure 2:**
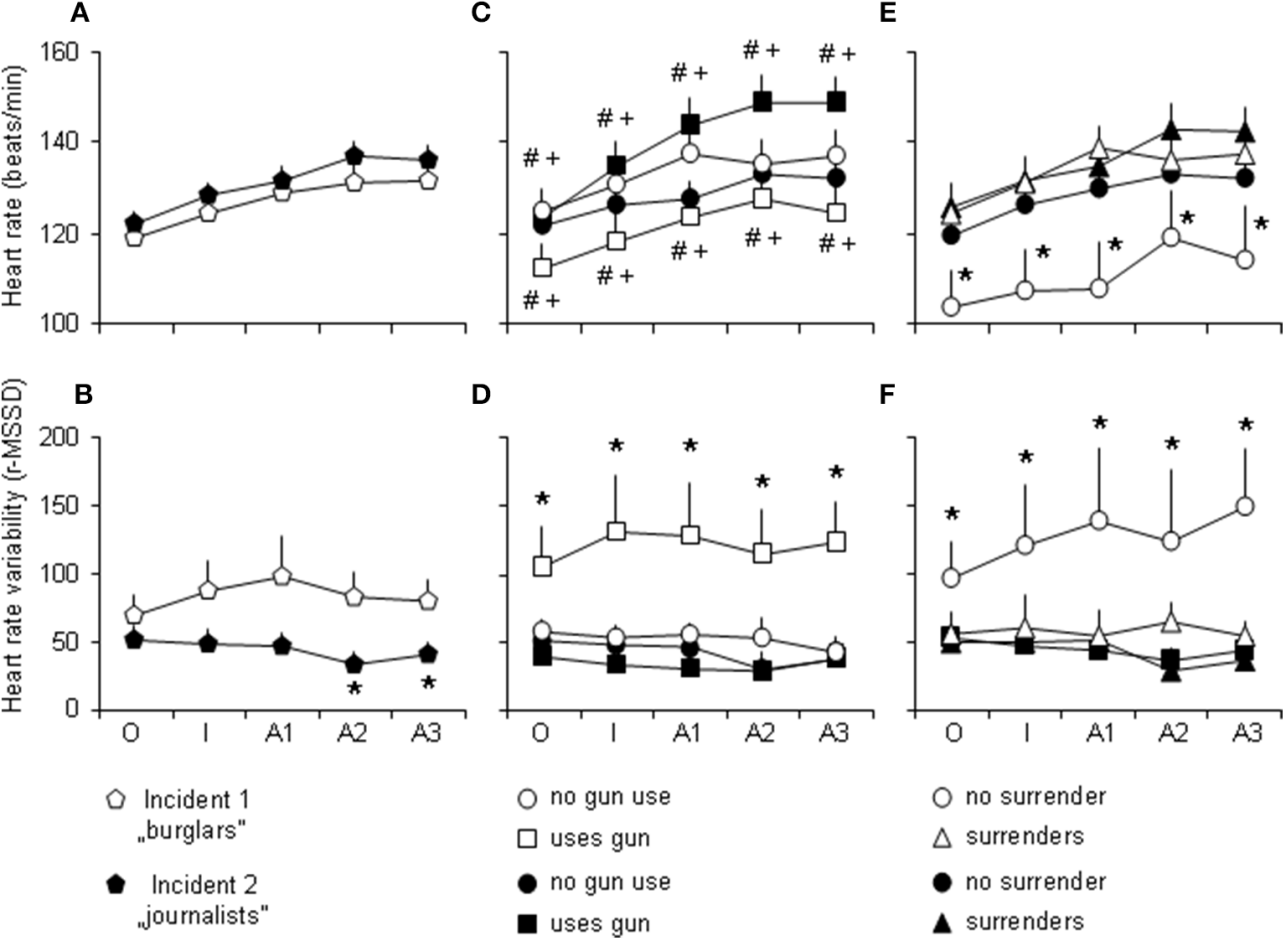
**Heart rates and heart rate variability grouped according to (A,B), the type of the incident, (C,D), gun use, and (E,F), surrendering**. O, the opening phase (previous to entering the flat); I, inspection, interrogation (non-aggressive period of the action phase); A1-3, min 1-3 of the aggression period of the action phase; r-MSSD, root mean of the squared successive differences (a measure of heart rate variability); ^*^, significantly different from all other groups; ^#^, significant differences between behavioral groups within the same incident;^+^, significant difference between gun using subjects of Incident 1 and 2 (*p* < 0.05 at least).

Both HR and HRV were relatively high by the start of incidents (Figure 2). HR was not affected by the type of the incident [*F*_incident (1, 114)_ = 1.57; *p* = 0.21; *F*_interaction (4, 456)_ = 1.15; *p* = 0.33], but significantly increased over the conflict [*F*_time (4, 456)_ = 23.39; *p* = 0.0001] (Figure 2A). The interaction between factors was highly significant in the case of HRV [*F*_interaction (4, 456)_ = 5.23; *p* = 0.0003] (Figure 2B). Particularly, Incident 2 was characterized by significantly lower heart rate variability during the aggression period of the action phase.

### Interactions between behavior and heart function

As indicated above, this analysis focused on gun use and surrender. Figures 2C–F show autonomic variables grouped according to gun use and surrendering, while statistics was presented in Table 2. An interesting characteristic of these findings is the minimal interaction between factor time and autonomic functions. HR increased over time in both comparisons, but no interactions with other factors were significant where factor time was considered. HRV did not change over time, and factor time showed no significant interaction with other factors. The interaction between factors “behavior” and “incident” was significant in all comparisons (Table 2), and revealed the following: gun use was associated with relatively low HR and high HRV in Incident 1, while in Incident 2, high HR and low HRV were observed in gun users. Surrender was associated with high HR and low HRV in Incident 1, while in Incident 2, HR and HRV did not differentiate surrendering and not surrendering officers. Thus, similar behaviors (gun use and surrendering) were associated with markedly different heart activity profiles in the two incidents. The lack of interaction with time suggests that autonomic changes were not consequences of aggressive behavior, because differences were present already in the opening phase and remained constant throughout.

**Table 2:** **The interaction between autonomic functions and behavior—statistics**.

**Interactions between factors “behavior” and “incident”**			
HR	Gun use	*F*_behavior ^*^ incident (1, 560)_ = 24.37	*p* < 0.00001
	Surrendering	*F*_behavior ^*^ incident (1, 560)_ = 7.57	*p* = 0.006
HRV (r-MSSD)	Gun use	*F*_behavior ^*^ incident (1, 560)_ = 15.13	*p* = 0.00012
	Surrendering	*F*_behavior ^*^ incident (1, 560)_ = 23.69	*p* < 0.00001
**Effects of time**			
HR	Gun use	*F*_time (4, 560)_ = 6.58	*p* = 0.0004
	Surrender	*F*_time (4, 560)_ = 4.44	*p* = 0.0015
HRV (r-MSSD)	Gun use	*F*_time (4, 560)_ = 0.05	*p* = 0.6
	Surrender	*F*_time (4, 560)_ = 0.89	*p* = 0.5
**Interactions between factors time, behavior and incident**			
HR	Gun use	*F*_time ^*^ incident (4, 560)_ = 0.50	*p* = 0.7
		*F*_time ^*^ behavior (4, 560)_ = 0.55	*p* = 0.7
		*F*_time ^*^ behavior ^*^ incident (4, 560)_ = 0.49	*p* = 0.7
	Surrender	*F*_time ^*^ incident (4, 560)_ = 0.16	*p* = 0.9
		*F*_time ^*^ behavior (4, 560)_ = 0.19	*p* = 0.9
		*F*_time ^*^ behavior ^*^ incident (4, 560)_ = 0.60	*p* = 0.7
HRV (r-MSSD)	Gun use	*F*_time ^*^ incident (4, 560)_ = 0.46	*p* = 0.8
		*F*_time ^*^ behavior (4, 560)_ = 0.15	*p* = 0.9
		*F*_time ^*^ behavior ^*^ incident (4, 560)_ = 0.44	*p* = 0.8
	Surrender	*F*_time ^*^ incident (4, 560)_ = 1.01	*p* = 0.4
		*F*_time ^*^ behavior (4, 560)_ = 0.91	*p* = 0.5
		*F*_time ^*^ behavior ^*^ incident (4, 560)_ = 1.47	*p* = 0.2

*The table shows ANOVA statistics for the findings presented in Figures 2C–F. HR, heart rates; HRV, heart rate variability; r-MSSD, root mean of the squared successive differences (a measure of HRV)*.

**Figure 3:**
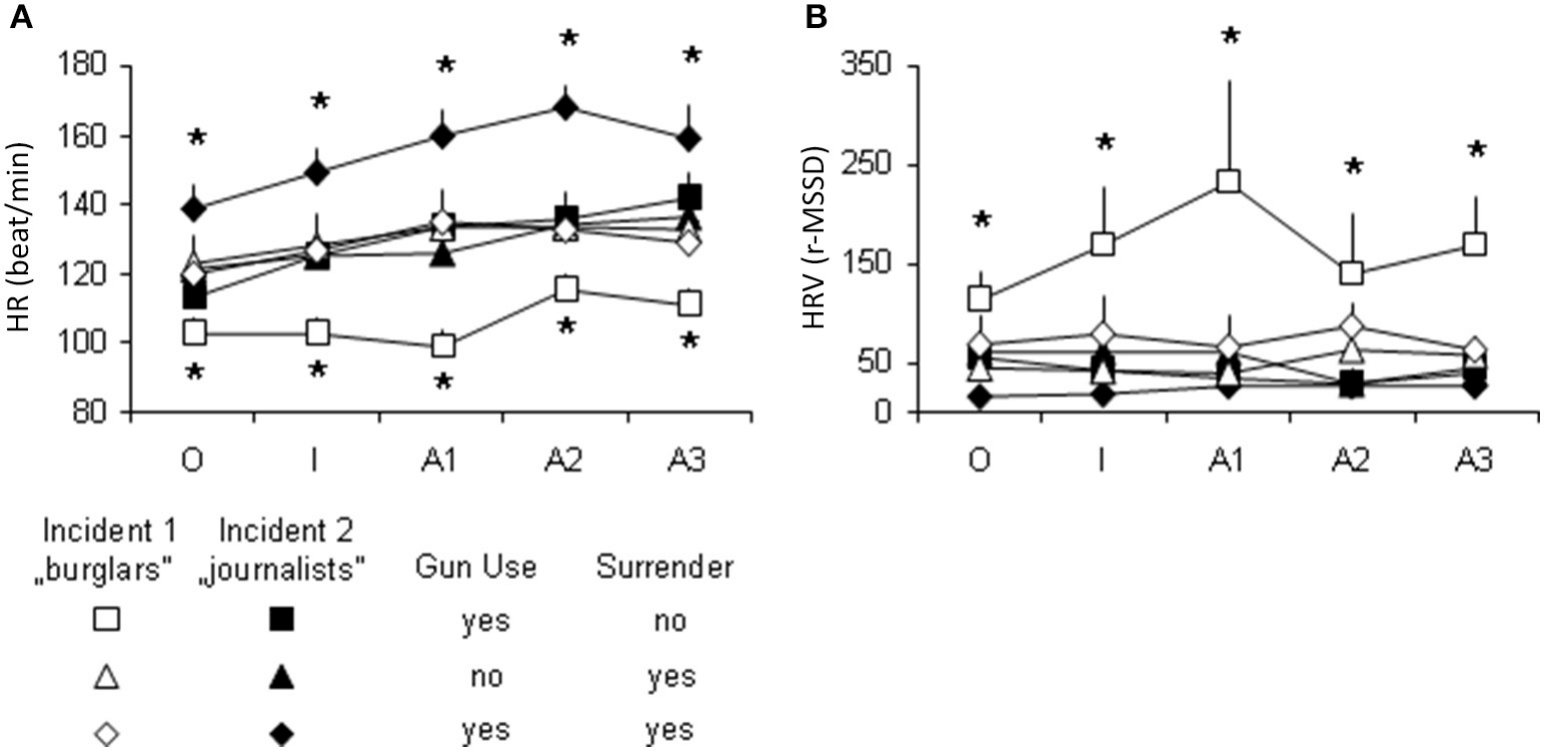
**Autonomic functions in subjects grouped according to gun use and surrendering shown in various combinations**. For the absence of the 4th group (no gun use, no surrender) see text. **(A)**, Heart rates (HR); **(B)**, Heart rate variability (HRV); r-MSSD, root mean of the squared successive differences (a measure of HRV); ^*^, significantly different from all the other groups (*p* < 0.05 at least).

In a second analysis we combined the two behaviors, and analyzed heart function in the following groups: gun use without surrendering; surrendering without gun use, and surrendering associated with gun use. The theoretically possible fourth group (neither gun use nor surrendering) was absent in Incident 1. This group was omitted from analysis, because this study aimed at comparing the two incidents. Again, factor time had little impact on autonomic functions. HR increased over time [*F*_time (4, 410)_ = 5.71, *p* = 0.00017], but this showed no interaction with other factors [*F*_time ^*^ incident (4, 410)_ = 0.70, *p* = 0.6; *F*_time ^*^ behavior (4, 410)_ = 0.32, *p* = 0.9; *F*_time ^*^ behavior ^*^ incident (4, 410)_ = 0.35, *p* = 0.9] (Figures 3A,B). HRV was not changed over time [*F*_time (4, 410)_ = 0.22, *p* = 0.9], and factor time interacted neither with incident nor with behavior [*F*_time ^*^ incident(4, 410)_ = 0.38, *p* = 0.8; *F*_time ^*^ behavior (4, 410)_ = 0.27, *p* = 0.9; *F*_time ^*^ behavior ^*^ incident (4, 410)_ = 0.61, *p* = 0.8]. This again suggests that group differences in autonomic functions were unrelated to the evolution of events within incidents. By contrast, the interaction between the incident and behavior was highly significant [*F*_behavior ^*^ incident (2,410)_ = 10.08, *p* = 0.00005]. This analysis differentiated two groups from the rest. Gun use without surrendering was associated with low HR and high HRV in Incident 1, while gun use followed by surrendering was associated with high HR and low HRV in Incident 2. According to the Multiple Regression analysis performed, the combination of gun use and surrender was predicted significantly by HR and HRV [*R* = 0.576; *F*_(11, 104)_ = 4.71; *p* = 0.0001], and explained 33.2% of behavioral variance. In this analysis, the criterion variables were the presence of gun use, surrender or both, while the predictor variables were HR and HRV measured across the 5 time-points of the incidents.

## Discussion

We found that under highly stressful conditions, decisions and behavior are primarily governed by arousal-related precursors of, and less by information gathered during the conflict. Particular behavioral profiles were associated with specific autonomic responses. e.g., unprovoked gun use combined with submission readiness under pressure was associated with high HR and low HRV, while refraining from the initiation of aggression combined with reluctance to surrender was associated with an opposite pattern of autonomic functions. Individual characteristics had a large impact on the interaction between autonomic functions and behavior, as cardiac profiles associated with particular behavioral responses were observed already at the beginning of social encounters i.e., before aggressive acts were initiated.

There were several decision points in both incidents that should have guided the behavior of officers. The circumstances of Incident 1 (burglars) indicated that a criminal offense is being committed. As the number of offenders was unclear—the flat was never searched thoroughly, consequently the sufficiency of police force was doubtful—the officers should have left the flat, secured the entrance and asked for reinforcement. After falling victim to the ambush prepared by suspects, the proper decision was surrender. Although gun use is theoretically not prohibited, circumstances made resistance hopeless. Under the circumstances of Incident 2 (journalists), criminal offense was not presumable. Consequently, the police intervention should have been limited to clear up the situation. Officers made wrong judgments at each decision point. In Incident 1, they disregarded the signs of danger and instead of asking for reinforcement continued the operation. When attacked, a considerable share of officers preferred fighting over surrender. In Incident 2, officers disregarded information that excluded the perpetration of criminal offenses by suspects and all initiated fights, despite the fact that physical aggression and gun use are prohibited under similar circumstances. The trainer's experience shows that even practiced officers take wrong decisions if not prepared for extreme stress situations.

We hypothesize that the first decision error—i.e., acting against information available in the training arena—was due to the high levels of physiological arousal (high HR) shown by all subjects at the start of incidents. It was reported rather early and amply confirmed later on that high arousal (high HR) reduces the cognitive complexity of perceptions and limit the capacity of noticing cues peripheral to central tasks (Easterbrook, 1959; Paulhus and Lim, 1994; Chajut and Algom, 2003). This phenomenon predicted by the Yerkes-Dodson law (Yerkes and Dodson, 1908) and later conceptualized as “attentional myopia” (Mann and Ward, 2004) was recently shown to be relevant for aggression control (Ward et al., 2008). A similar role was attributed to HRV (Park et al., 2013), which was also high in certain categories of subjects. We hypothesize that “attentional myopia” prevented officers from noticing or considering cues suggestive of danger (Incident 1 “burglars”) or those prohibiting the use of force (Incident 2 “journalists”).

The second decision error—i.e., hopeless resistance in Incident 1 and unprovoked aggression in Incident 2—appears to be related to particular HR and HRV profiles. A growing body of evidence suggests that people with high HRV—as compared to those showing low HRV—perform better in tests of cognitive performance, show flexible emotional and behavioral responses, recognize the emotions of partners better, and appear to be more able to control aggressive tendencies (Hansen et al., 2003; Ruiz-Padial et al., 2003; Ivarsson et al., 2009; Thayer et al., 2009; Quintana et al., 2012). Recently, Park and Thayer (2014) put forward the idea that HRV may be the peripheral (vagus-mediated) reflection of central decision-making processes, particularly of prefrontal-subcortical inhibitory functions. According to this theory, the suppression of phasic HRV (via the downregulation of the cardiac vagal tone) signals the impairment of central inhibitory mechanisms, while increased HRV signals self-regulatory efforts. Based on these findings together with those showing that high HR is associated with a bias toward hostile attributions and aggression (Williams et al., 2003; Lorber, 2004; Patrick, 2008), we suggest that increased HR promoted hostility and aggression proneness in both incidents, but these predilections were materialized in the second incident only, where prefrontal-subcortical inhibitory circuits involved in decision making and aggression were disrupted as shown by the suppression of HRV. Taken together, these findings suggest that inappropriate aggression (Incident 2) develops when HR is high (tentatively resulting in both “attentional myopia” and enhanced aggression-proneness), and HRV is low, which signals diminished self-control.

An interesting pattern of physiological changes was observed in Incident 1, where the most inappropriate behavior (gun use without surrender) was associated with the lowest HR and the highest of HRV noticed in this study. The interpretation of this finding is difficult at present. In Incident 2, the same behavioral profile was associated with significantly higher HR and lower HRV, suggesting that autonomic responses were not particularly related to the behaviors performed. One may assume that these discrepant autonomic responses were related to the nature of the aggressive interaction, as provoked (Incident 1) and non-provoked aggression (Incident 2) can be considered analogous to reactive and proactive aggressions, respectively. Our findings, however, are at variance with earlier assumptions on the association between these types of aggression and autonomic functions. It was suggested that reactive aggression is associated with high HR and low HRV (Pico-Alfonso et al., 2007; Scarpa et al., 2010; Murray-Close and Rellini, 2012), while in our sample provoked aggression was associated with low HR and high HRV (Incident 1). In the same vein, proactive aggression is supposed to correlate with low HR and high HRV, while in our sample non-provoked (proactive) aggression was associated with high HR and low HRV (Incident 2). The fundamental differences between the models used earlier and the ones used here may be one of the possible explanations for these discrepancies. Previous hypotheses were either based on models where the perpetration of real aggressive acts and the measurement of autonomic activity were temporally detached (Scarpa et al., 2010), or where the aggressiveness of, or submission by, subjects was symbolic or mild (Pico-Alfonso et al., 2007; Murray-Close and Rellini, 2012). One can hypothesize that the association of aggressiveness and autonomic functions is different when the latter are recorded in parallel with real acts of aggression. Alternatively, the unexpected association of behavioral and cardiac responses is explained by the cardiac concomitants of decision-making rather than by behavior. As shown above, findings obtained in Incident 2 are consistent with current theories on the autonomic correlates of aggression-related cognitive functions. One can tentatively assume that the same is valid for Incident 1 where resilience in a hopeless situation may be considered a symptom of self-regulatory effort (expected to correlate with high HRV) in the meaning that officers resisted the temptation of surrendering i.e., they self-regulated themselves into perseverance. Irrespective of the validity of these alternative hypotheses, our findings show that the interaction between autonomic responses, decision-making and aggression is more complex than previously thought, and warrants further studies.

An interesting feature of our findings is that autonomic responses depended to a large extent on intrinsic subject characteristics. Although HR increased over the conflict, and HRV changes were incident-dependent overall, those subjects of Incident 1, who used their guns without surrendering showed low HR and high HRV before these behaviors were actually displayed. Similarly, those subjects of Incident 2 who used their guns without provocation but surrendered later on showed high HR and low HRV already in the opening phase. This finding appears important in two respects. Firstly, animal studies suggest that heart rate dynamics is slower than the decision-making process (Nephew et al., 2003; Tovote et al., 2005), which may weaken our assumptions on the causality of cardiac autonomic changes. Nevertheless, situation- and behavior-typical autonomic profiles were observed already at the start of incidents which ensures the time necessary for cardiac responses to influence decisions. Secondly, personality factors strongly affect motivational states, autonomic responses, aggressiveness and their relationship under stressful conditions (Taylor, 1967; Humphreys and Revelle, 1984; Zellars et al., 2009; Brumbaugh et al., 2013). As such, our findings suggest that while the circumstances of the conflict do affect autonomic functions, these also depend on personality differences in a context-dependent manner, and as such may be used as predictors of aggression- and resilience-proneness under conditions of stressful social interactions.

### Limitations

This was an observational study, implying that the interests of the training prevailed. Due to time constraints, subjects were poorly characterized regarding their personality and psychological features, antecedents etc. The homogeneity of the sample may partly compensate for this deficiency; all subjects were physically and psychologically healthy police officers. Nevertheless, future studies need to address these issues in order to evaluate the impact of social and psychological factors omitted here. Another limitation is that the equipment used to measure autonomic activity lacks the details provided by ECG recordings. Future studies using more sophisticated equipment may unravel aspects of cardiac activity that remained hidden to this study. Finally, the recording of basal autonomic functions together with the assessment of basal and training-related changes of other stress responses (e.g., plasma cortisol) may considerably increase the scope of future studies.

### Implications

Although performed within the framework of a training process, the subjects of this study performed genuine acts of aggression and violence. As such, the training system used in this study combines the “virtues” of the two main approaches employed so far in human aggression research: it allows the study of biological correlates in parallel with the actual execution of real aggressive acts (for the two categories of approaches see Introduction and Haller, 2014).

The present study demonstrates that under highly stressful conditions, decisions and behavior are primarily governed by the precursors of the critical situation and less by information gathered during the conflict. It also suggests that intrinsic characteristics reflected by cardiac autonomic measures have a large impact on the interaction between psychological precursors and behavior. We suggest that the realistic nature of the model opens new perspectives for studying the complex interaction between emotional antecedents, decision making and behavior during social conflict.

## Author contributions

Corresponding author Jozsef Haller designed behavioral analysis and wrote the paper. Authors Gabriella Raczkevy-Deak and Katalin P. Gyimesine performed behavioral analysis. Authors Andras Szakmary, Istvan Farkas, and Jozsef Vegh designed the training procedure and performed the study.

### Conflict of interest statement

The authors declare that the research was conducted in the absence of any commercial or financial relationships that could be construed as a potential conflict of interest.
